# Association of Safe Disposal of Child Feces and Reported Diarrhea in Indonesia: Need for Stronger Focus on a Neglected Risk

**DOI:** 10.3390/ijerph13030310

**Published:** 2016-03-11

**Authors:** Aidan A. Cronin, Susy Katikana Sebayang, Harriet Torlesse, Robin Nandy

**Affiliations:** 1 UNICEF Indonesia, World Trade Center 6, Jalan Jenderal Sudirman Kav. 31, Jakarta 12920, Indonesia; htorlesse@unicef.org (H.T.); rnandy@unicef.org (R.N.); 2 Faculty of Public Health, Universitas Airlangga, Banyuwangi Campus, Jalan Wijaya Kusuma No 113, Banyuwangi 68414, Indonesia; sksebayang@gmail.com

**Keywords:** Indonesia, child feces disposal, diarrhea, water, sanitation, hygiene, care practices

## Abstract

Indonesia still faces several challenges in the areas of water, sanitation, and hygiene (WASH). Diarrhea remains a major killer of children and it is important to understand the local diarrhea transmission pathways to prioritise appropriate WASH interventions to reduce diarrhea burden. This study used a cross-sectional data set from a recent national household survey (the 2012 Indonesia Demographic and Health Survey) to examine the associations between diarrhea in children aged less than 24 months with WASH interventions and population characteristics. Unsafe disposal of child feces was strongly associated with an increased odds of child diarrhea (OR: 1.46; 95% CI: 1.18–1.82, *p* = 0.001). However, other WASH practices were not found to be associated. The findings underline the dangers of unsafe disposal of child feces and highlight the need for strengthening the related policies and program strategies and their implementation.

## 1. Introduction

Efforts to improve water, sanitation and hygiene (WASH) have been accelerated in Indonesia, as part of the overall drive to achieve the Millennium Development Goals, resulting in accelerated progress on WASH access [1]. Indonesia is reported to have met the MDG target for increased population access to an improved water source but is not on track to achieve sanitation targets [2]. Indeed, open defecation, which is practiced by an estimated 51 million people in Indonesia, remains a major challenge [2]. This is the second largest country burden of open defecation in the world, after India. Every year between 136,000 and 190,000 under five children die in Indonesia [3], primarily from preventable causes with diarrhea and pneumonia likely responsible for more than 40% of these deaths. Diarrhea remains a major contributor to child mortality, accounting for an estimated 31% of deaths among children aged one year and 25% of deaths among children aged 1–4 years [4]. 

Globally, up to 88% of the diarrhea disease burden has been estimated to be linked to incomplete water, sanitation and hygiene provision, with the vast majority occurring in children in developing countries [5]. A more recent meta-analysis on WASH and diarrhea concluded that improvements to both water and sanitation can result in large potential reductions in diarrheal disease risk in low- and middle-income settings [6]. It is important to understand the local diarrhea transmission pathways, especially in children, in order to prioritize the appropriate WASH strategies and interventions, including efforts towards appropriate behavior change in order to cut diarrhea incidence; for example the Government of India’s national WASH behavior change communication strategy prioritizes four key interventions to break fecal-oral transmission: toilet usage, handwashing with soap, consumption of safe water and proper disposal of child feces [7]. The first three routes have been examined to some extent in the Indonesian context [8,9,10,11,12,13], as well as foodborne diarrhea [14,15] but the issue of proper disposal of child feces has received insufficient attention so far, despite this being recognized as a key risk, even when latrines are available [16]. 

The objective of this study was to identify the key WASH indicators associated with diarrhea among Indonesian children aged less than 24 months and to determine possible subgroups in the population needing programmatic focus given the importance of this period for child health and nutrition. In particular it explores the potential roles and extent of unsafe disposal of child feces on diarrhea transmission in the county.

## 2. Materials and Methods 

Data from the 2012 Indonesia Demographic Health Survey (IDHS) were used in the analysis [17]. The IDHS, a large scale household survey, was designed to provide representative data at the national and provincial level disaggregated by urban and rural locations. The survey methodology and scope was approved by an ethics review panel [17]. 

The primary focus of this analysis was to identify determinants of reported diarrhea in the two weeks prior to the survey among children aged below 24 months. In households with more than one child aged below 24 months, only the youngest child was included in the analysis as data on infant and young child feeding (IYCF) practices, antenatal care during most recent pregnancy and delivery care are likely to be more accurate and readily available for the last child. Children aged less than 24 months were chosen because of the increased risks associated with diarrhea and, due to their exposure and vulnerability, to multiple transmission routes. 

The analysis was undertaken using the dataset with the complete set of WASH indicators and other covariates. WASH indicators included household sanitary facility, source of household drinking water, reported household water treatment, availability of soap for handwashing in the household, reported use of soap for handwashing, and reported disposal of child’s feces. Covariates included child’s age and sex; mother’s perception of the size of her child at birth; IYCF practices; mother’s education and age; father’s education and age; household wealth quintile (based on an asset index); number of household members; number of antenatal care visits during last pregnancy; assisted delivery; birth in facility and woman’s participation in household decisions (on wealth, large household purchases, family visits and expenditure). Mother’s education level was categorized into primary or no education, secondary school, and higher. 

The World Health Organization’s definitions for indicators of IYCF practices in children aged less than 24 months were applied [18]. In addition, a variable on “age-appropriate feeding” that combined exclusive breastfeeding for children aged 0–5 months with minimum acceptable diet for children aged 6–23 month was determined; a child was considered to have age-appropriate feeding if she/he was aged 0–5 months and exclusively breastfed, or aged 6–23 months and consumed a minimal acceptable diet.

Improved household water sources included piped water into a dwelling; piped into a yard or plot; public tap; protected well in a dwelling; protected well in a yard or plot; protected public well, and rainwater, in line with the global Joint Monitoring Protocol (JMP) methodology [2]. Treated water included boiling, bleaching, filtering, and solar disinfecting. A pour-flush toilet with septic tank was considered as improved sanitation. For hygiene indicators, observed availability of soap, use of soap for handwashing and safe disposal of child’s feces were analyzed.

Safe disposal of child’s feces implies there is minimal risk of the excreta entering fecal-oral pathogen transmission pathways and is only possible where there is access to an improved toilet or latrine. Thus, “improved child feces disposal” is defined when a child’s feces is discarded or rinsed into an “improved” toilet or latrine [19,20]. Methods of disposal of child feces that are termed unsafe in this analysis include feces being thrown outside the dwelling, left in the open, buried in the yard, and rinsed away into anything but an improved toilet or latrine. In addition, the usage of all types of diapers (washable or disposable) is regarded as unsafe given that the current solid waste disposal practices in Indonesia can not systematically ensure the safe containment of excreta; the national sanitation program also addresses this gap [21]. 

Variables with a *p*-value of <0.25 in univariate analysis were included in the multivariate analyses. Variables with *p*-value of <0.05 after backward elimination were retained in the final model. Logistic regression was used to estimate the odds ratio for diarrhea in the last two weeks; prevalence ratios are provided in supplementary tables for comparison purposes. Clustering, sampling stratification and sampling weights were taken into account using the svyset command in Stata 11. Pre-determined sub-group analyses were also undertaken to investigate the possible groups needing extra programmatic attention. The subgroups were sex of child, and birth size to represent child’s biology, woman’s education to represent socio economic status, and other WASH indicators that potentially interact with fecal disposal (sanitation, water source and soap availability). For the subgroup analysis, woman’s education was categorized as having higher education or not and birthsize was categorized as birthweight less than average or not. Interaction tests were used to assess the statistical difference in association between subgroups. Interactions were considered significant for *p*-values < 0.01. 

## 3. Results

There were 4909 observations with data on diarrhea, WASH indicators, and covariates out of 6778 observations for children under 24 months. Compared to all children under 24 months, the children in the dataset were slightly better off in terms of sanitation (improved sanitation: 69% *vs.* 61%), water source (improved water: 62% *vs.* 59%), optimal feeding practices (age appropriate feeding: 37% *vs.* 34%), socio-economic status (highest wealth quintile: 23% *vs.* 19%; higher education for women: 16% *vs.* 13%, higher education for husband: 14% *vs.* 12%), and mothers pregnancy care and delivery (ANC visit ≥ 4: 91% *vs.* 88%; assisted delivery: 90% *vs.* 85%). 

Table 1 presents the background characteristics on the children, their mothers and households. Fifty-two percent of children were boys and 52% were aged less than one year. One in eight (12.4%) mothers reported that their children were smaller than average or of very small size at birth. The majority of mothers had secondary education (57%), were aged 20 to 29 years (52%) and had high participation in key household decisions (all over 80%). Though access to improved water and sanitation was relatively high in this population (62% and 69% respectively) only 47% practiced safe disposal of child feces (Table 1). Availability of soap for handwashing was very high at 94%. Table 2 presents the risk factors associated with diarrhea. The bivariate analysis shows a number of significant associations with diarrhea, including mother’s age (<20), husband’s age (<30), mother and husband’s level of education (primary or less), lack of household decision-making by the mothers, lowest wealth quintile, and perceived size of the child at birth (as very small) and children aged over 12 months. Though the odds of diarrhea were significantly lower among children living in households with improved sanitation, in addition to improved water source, availability of soap and reported use of untreated water was also associated with lower odds of diarrhea. The odds of diarrhea is significantly greater in households practicing unsafe disposal of child feces than those practicing safe disposal (OR: 1.37; 95% CI:1.11–1.69, *p* ≤ 0.0001).

The multivariate analyses indicated that unsafe disposal of a child’s feces, child’s age, reported birth size, woman’s education, woman’s age, and lack of mother’s participation in decision-making of her own health remained significantly associated with diarrhea. Adjusting for covariates, disposal of child feces was significantly associated with an increased odds of diarrhea (OR: 1.46; 95% CI: 1.18–1.82, *p* = 0.001) (Table 2). Understanding that the urban and rural disparity may be important, we also did the analysis using rural/urban disaggregation and found that this variable was not significant in the multivariate nor the subgroup analysis.

Table 3 shows the results of subgroup analysis that examined interactions between specific determinants, controlling for key factors. After adjusting for child’s age, reported birth size, woman’s education, woman’s age, and woman participation in decision of her own health, there is a tendency for interaction with gender where the odds of diarrhea associated with unsafe disposal were greater in female children compared to boys (OR: 1.93; 95% CI: 1.41–2.65, *p* < 0.0001, *p* for interaction = 0.02).

In addition, there is a tendency that the association between unsafe disposal and diarrhea was greater if the household had unimproved sanitation (OR: 2.19; 95% CI: 1.40–3.43, *p* = 0.001, *p* for interaction = 0.04)). None of the interactions for the other variables nor the Interaction P values were significant. (Table 3).

## 4. Discussion

Our analysis of the IDHS 2012 dataset showed that the odds on diarrhea in children aged less than 24 months were significantly higher among children living in households that practiced unsafe disposal of child’s feces compared with those that practice safe disposal after adjusting for other WASH indicators and covariates. We did not find an association between sanitation or access to improved water and diarrhea, unlike that reported from other international research e.g., [22] or within Indonesia e.g., [12]. We also did not find any association between soap availability or improved water quality with diarrhea as might be expected. For example, a community-based water treatment kiosk scheme in urban slums in northern Jakarta reduced diarrhea in children aged 6–36 months by 49% (RR: 0.49; 95% CI: 0.29–0.83) [9]. 

Our findings are consistent with a meta-analysis of 10 observational studies from across several countries and found that child feces disposal behaviors considered risky (open defecation, stool disposed of/or visible in the open) were associated with a 23% increase in risk of diarrheal diseases (RR: 1.23, 95% CI: 1.15–1.32) while behaviors considered as safe (use of latrines, nappies, potties, toilets, washing diapers) were borderline protective (RR: 0.93, 95% CI: 0.86–1.00) [23]. Other identified factors, as identified from analyses of DHS studies examing child diarrhea and nutrition indicators have highlighted, for example, the importance of distance to water source [24] and livestock ownership [25] which were not factored into this study. 

Using DHS data for Northwest Ethiopia, it was found that improper child stool disposal along with low level of maternal education; lack of toilet; having more than two under five children; higher birth order and age of children were risk factors for childhood diarrhea after adjusting for other variables [26]. Similarly, a multivariate analysis from Iraq found that diarrhea was associated with age of child, area of residence, maternal education, source of water, toilet facility, unsafe disposal of children's stool and improper disposal of dirty water [27]. 

The association found in our analyses between unsafe disposal of child feces and diarrhea alone, but not sanitation or soap availability or feeding practices, therefore, differs with much of the literature. However, the subgroup analyses did find a tendency for interaction with unimproved sanitation (Table 3). A combination of both the IDHS 2007 and 2012 datasets found piped water; child age and sex; household wealth; living in an urban area; sanitation coverage; health status and health facilities to be associated with diarrhea incidence while water treatment, and mother’s education were not significantly associated with diarrhea; that study did not look at safe child feces disposal [13]. While that study employed two datasets and looked at children under age 5, there are common findings with this analysis for water treatment and the type of toilet at household level. 

Potential reasons for the lack of association of WASH factors with diarrhea, apart from safe disposal of children’s feces, may be due to the unique contaminant exposure pathways to which children under the age of two years are exposed, such as the importance of direct fecal contamination of child’s hands or play areas, poor hygiene practices of caregivers or geophagy [28,29]. This may also be influenced by local context-specific cultural and environmental factors. The authors have not controlled for other household members having diarrhea and so this is a possible limitation. The association between untreated water and lower odd of diarrhea may reflect on the poor protective effect of water treatment, as evidenced by significant frequency and levels of fecal contamination of stored boiled water in one Indonesian setting [30]. One other limitation is that, given the wealth quintiles provided in the IDHS dataset were used, these would have also included water and sanitation assets. 

We could not confirm the caregiver’s handwashing practice while the availability of soap at the place for hand washing place as a proxy for handwashing practice was not associated with diarrhea. Interestingly, we did find an association between maternal education (primary or less), age (under 20 years), and lack of participation in decision making with diarrhea, after adjusting for household wealth. 

Although sanitation is not independently associated with diarrhea in our study, the greater odds of diarrhea due to unsafe disposal of child feces in households with unimproved sanitation is consistent with the pathways between unimproved sanitation and diarrhea. The greater odds of diarrhea due to unsafe disposal of child feces, and diarrhea in girls compared to boys, could indicate a possibility of gender bias in cleaning infants. There may be a tendency to consider boys dirtier and, therefore, a need to clean them more thoroughly than girls. We could not, however, confirm this. The tendency for interaction between unsafe disposal of child feces and gender, as well as sanitation, indicate the need for further research to optimize programmatic focus, with this study providing some possible and logical points of intervention and to assess this within the broader context of gender differences in child health outcomes [31,32]. Other studies working with mothers to determine feasible and acceptable safe practices for safe disposal of child feces have shown encouraging results around behavior change [33,34,35], of particular importance in Indonesia given the impact of maternal education on child health outcomes [36,37]. Comprehensive reviews and frameworks of behavior change approaches may be employed to better inform this work at scale [38,39].

Due to smaller size and less smell, the feces of young children may be regarded by caregivers as less harmful than adults [23,40] though may possibly have a higher loading of pathogens [41,42]. Globally-insufficient analysis has been done on the child excreta disposal interventions, despite its potential importance as a high risk factor; safe disposal of children’s stools has generally not been an important component to date in sanitation programs [40]. A formative study in Eastern Indonesia found that 80% of respondents “disagreed” or “strongly disagreed” with the statement “Baby’s feces do not spread disease”, indicating a high level of knowledge that children’s feces is a health risk [43]. However, correct knowledge on the dangers of child feces did not always translate to safe child feces disposal practices; 48% of the same households with children aged less than five years indicated that they dispose of the child feces unsafely in the environment. These reported levels of safe disposal (52%) are lower than East Java where 66% reported safe disposal of child feces [44]. Another study from 3 Indonesian Districts, one each in Java, NTT, and Papua, found only 43.4% of household reporting safe child feces disposal [45], again indicating the extent of the problem. 

Recent advocacy emphasized that unsafe disposal practices are common even in households with improved sanitation [19]. Despite the inclusion of the concept of safe disposal of child feces in national guidance around open defecation-free communities [21], few WASH interventions in Indonesia specifically target the issue and so this has been, in the most part, a neglected intervention [19]. This is similar to findings from other countries, e.g., [46,47,48]. 

The findings from this analysis reinforce the association of unsafe disposal of child feces and child diarrhea. This underlines the importance of addressing this issue in policy formulation and program implementation [19,20]. The importance of household sanitation facilities is important, but not adequate, and the safe disposal of child feces is related to practice and behaviors. Appropriate behavior change interventions to address disposal of child feces will, therefore, need to be included systematically in the national community sanitation program. In addition, this message needs to be reinforced through the health care infrastructure particularly those working on maternal and child health, and on infant and young child feeding. In order for this to happen effectively, further formative research is still needed in Indonesia on better identification of enablers and barriers to behavior change of safe disposal of child feces. Formative studies in the context of Indonesia, e.g., [20,43] are especially critical to guide interventions given the diversity, population, and large geographic expanse of the country and also given the need to urgently foster greater demand for sanitation in Indonesia [49]. Further research may also examine a broader range of health and nutrition indicators [45], especially given the challenges with self-reporting of diarrhea [50]. 

This focus is in line with recent global calls and guidance for renewed action combining the hardware in support of WASH with the ‘software’ which addresses the required behavior change to optimize the impact of these services in communities and institutions towards the desired health impact [51]. Global guidance, such as [52], may also present opportunities to advocate stronger on this key issue. 

## 5. Conclusions 

In conclusion, our multivariate analysis examined risk factors for diarrhea in Indonesian children aged less than 24 months using data from the 2012 national IDHS survey and found a strong association between unsafe child faeces disposal practices and diarrhea, but no significant multivariate association with the source of drinking water, household water treatment or availability of soap for handwashing. These associations were independent of socio-economic status and other population characteristics, except for unimproved sanitation and child sex. 

These findings merit a stronger emphasis on the safe disposal of child feces in sanitation policy, implementation, and monitoring in sanitation programs in Indonesia with mothers being the critical entry point for behavior change. This could be an important component of broader efforts to accelerate WASH service provision at home and in institutions to improve maternal and newborn health and nutrition.

## Figures and Tables

**Table 1:** Population characteristics * of children <24 months in the Indonesian Demographic Health Survey 2012 (N = 4909).

Factors		Number (*N*)	Proportion (%)
Household Characteristics			
Sanitation	Unimproved	1537	31.3
Water source	Unimproved	1886	38.4
Water treatment	Untreated	1534	31.2
Availability of soap for hand washing	Unavailable	307	6.3
Safe disposal of child’s feces	Unsafe	2593	52.8
	Lowest	655	13.3
	Second	951	19.4
	Third	1031	21.0
	Fourth	1162	23.7
	Highest	1110	22.6
Child characteristics			
Sex	Boys	2548	51.9
	Girls	2362	48.1
Age of child	12–23 months	2364	48.1
	6–11 months	1321	26.9
	0–5 months	1225	24.9
Birth size **	Very small	64	1.3
	Smaller than average	546	11.1
	Average	2793	56.9
	Larger than average	1315	26.8
	Very large	192	3.9
Birth assisted by trained professionals	No	499	10.2
Birth in facility	No	2983	60.8
Age-appropriate feeding	No	3106	63.3
Women Characteristics			
Women's education	Primary or less	1342	27.3
	Secondary	2804	57.1
	Higher	763	15.5
Husband's education	Primary or less	1424	29.0
	Secondary	2789	56.8
	Higher	696	14.2
Woman's age	<20	277	5.6
	20–29	2566	52.3
	30–39	1857	37.8
	≥40	209	4.3
Husband's age	<30	1562	31.8
	30–39	2443	49.8
	≥40	904	18.4
Number of household members	>4	3114	63.4
Woman participates in decision on own health	No	734	15.0
Woman participates in decision on large household purchase	No	837	17.1
Woman participates in decision on visit to family	No	744	15.2
Woman participates in decision on what to do with money	No	535	10.9
Number of antenatal care visits received by mother during pregnancy	<4	418	8.5
	≥4	4492	91.5

* Complete dataset for sanitation, source of water, water treatment, availability of soap for handwashing, disposal of child’s feces, wealth quintile, woman’s education, gender, infant feeding practice, birth size, woman’s age, husbands’ age, husband’s education, number of household member, woman’s participation in decision-making, antenatal care, assisted delivery, delivery at facility, and age of child <24 months; ** Mother’s perception of birth size.

**Table 2:** Odd Ratio (OR) for risk factors for diarrhea in children aged 0–23 months in Indonesia (*N* = 4909).

Factors		Diarrhea (%)	N	Unadjusted (Bivariate)			Adjusted (Multivariate)		
				OR	95% CI	*p*	OR	95% CI	*p*
Sex	Boys	19.3%	2548	1.16	(0.93, 1.45)	0.19			
	Girls	17.1%	2362	Ref					
Age of child (months)	12–23	20.6%	2364	1.77	(1.34, 2.34)	0.0003	2.15	(1.61, 2.87)	<0.0001
	6–11	19.2%	1321	1.62	(1.19, 2.22)		1.85	(1.35, 2.53)	
	0–5	12.8%	1225	Ref			Ref		
Mother’s perception of child size at birth	Very small	43.1%	64	4.96	(1.77, 13.91)	0.017	5.02	(1.73, 14.71)	0.033
	Smaller than average	19.8%	546	1.62	(0.75, 3.51)		1.63	(0.75, 3.52)	
	Average	18.2%	2793	1.46	(0.73, 2.95)		1.50	(0.74, 3.03)	
	Larger than average	17.1%	1315	1.35	(0.66, 2.77)		1.40	(0.68, 2.87)	
	Very large	13.2%	192	Ref			Ref		
Age-appropriate feeding	No	18.1%	3106	0.97	(0.78, 1.21)	0.79			
	Yes	18.5%	1803	Ref					
Woman’s age (years)	<20	29.0%	277	2.42	(1.21, 4.84)	<0.0001	2.37	(1.17, 4.80)	0.0001
	20–29	20.3%	2566	1.51	(0.87, 2.62)		1.50	(0.86, 2.62)	
	30–39	14.1%	1857	0.98	(0.56, 1.71)		0.97	(0.55, 1.71)	
	≥40	14.5%	209	Ref			Ref		
Husband’s age (years)	<30	23.7%	1562	1.81	(1.31, 2.51)	<0.0001			
	30–39	16.1%	2443	1.13	(0.83, 1.52)				
	≥40	14.6%	904	Ref					
Woman’s education	Primary or less	19.4%	1342	1.77	(1.19, 2.64)	0.0087	1.60	(1.08, 2.38)	0.04
	Secondary	19.4%	2804	1.76	(1.22, 2.54)		1.63	(1.12, 2.37)	
	Higher	12.0%	763	Ref			Ref		
Husband's education	Primary or less	20.6%	1424	1.92	(1.26, 2.92)	0.0095			
	Secondary	18.6%	2789	1.70	(1.13, 2.55)				
	Higher	11.9%	696	Ref					
Number of household members	>4	19.1%	3114	1.16	(0.93, 1.45)	0.18			
	≤4	16.8%	1795	Ref					
Wealth quintile	Lowest	22.9%	655	1.89	(1.32, 2.71)	0.013			
	Second	19.8%	951	1.57	(1.09, 2.25)				
	Third	19.3%	1031	1.51	(1.03, 2.21)				
	Fourth	17.8%	1162	1.37	(0.93, 2.02)				
	Highest	13.6%	1110	Ref					
Sanitation	Unimproved	20.7%	1537	1.27	(1.01, 1.60)	0.05			
	Improved	17.1%	3372	Ref					
Safe disposal of child’s feces	Unsafe	20.4%	2593	1.37	(1.11, 1.69)	<0.0001	1.46	(1.18, 1.82)	0.001
	Safe	15.8%	2316	Ref					
Availability of soap for hand washing	Unavailable	24.9%	307	1.53	(1.09, 2.15)	0.01			
	Available	17.8%	4602	Ref					
Water source	Unimproved	19.1%	1886	1.10	(0.89, 1.36)	0.37			
	Improved	17.7%	3023	Ref					
Water treatment	Untreated	17.5%	1534	0.93	(0.74, 1.17)	0.54			
	Treated	18.6%	3375	Ref					
Number of ANC visits of mother during pregnancy	<4	22.2%	418	1.31	(0.97, 1.77)	0.08			
	≥4	17.9%	4492	Ref					
Birth assisted by trained professionals	No	20.4%	499	1.17	(0.88, 1.56)	0.29			
	Yes	18.0%	4410	Ref					
Birth in facility	No	19.2%	2983	1.17	(0.92, 1.48)	0.19			
	Yes	16.8%	1926	Ref					
Woman participates in decision on own health	No	24.3%	734	1.55	(1.16, 2.07)	0.003	1.45	(1.09, 1,91)	0.01
	Yes	17.2%	4175	Ref			Ref		
Woman participates in decision on large household purchase	No	22.2%	837	1.36	(1.05, 1.75)	0.02			
	Yes	17.4%	4072	Ref					
Woman participates in decision on visit to family	No	23.1%	744	1.42	(1.10, 1.85)	0.01			
	Yes	17.4%	4165	Ref					
Woman participates in decision on what to do with money	No	21.8%	535	1.29	(0.94, 1.78)	0.12			
	Yes	17.8%	4374	Ref					

**Table 3:** Subgroup analysis on association between diarrhea in children aged less than 24 months and disposal of child’s feces by biological indicators, socio economic indicators and WASH indicators.

Subgroup	N	Adjusted (Multivariate) *				
		OR	95% CI		*p*	Interaction *p*
			Lower	Upper		
Biological Indicator						
Gender *						
Male	2548	1.15	0.85	1.56	0.37	0.02
Female	2362	1.93	1.41	2.65	<0.0001	
Birth Size **						
Smaller than Average	610	2.31	1.27	4.22	0.006	0.12
Average to Very Large	4299	1.38	1.09	1.74	0.007	
Socio Economic Indicator						
Woman’s education ***						
secondary or lower	4146	1.52	1.20	1.92	<0.0001	0.28
Higher	763	1.08	0.62	1.89	0.79	
Sanitation*						
Unimproved	1537	2.19	1.40	3.43	0.001	0.04
Improved	3372	1.24	0.94	1.62	0.12	
Water Source *						
Unimproved water	1886	1.59	1.12	2.23	0.009	0.57
Improved water	3023	1.39	1.06	1.84	0.018	
Soap Availability *						
No	307	1.32	0.61	2.83	0.479	0.81
Yes	4602	1.45	1.16	1.82	0.001	

* Adjusted for woman’s education, child’s age, birth size, woman’s age, and woman participation in decision of her own health; ** Adjusted for woman's education, child’s age, woman’s age, and woman participation in decision of her own health; *** Adjusted for child’s age, birth size, woman’s age, and woman participation in decision of her own health.

## Supplementary Materials

The following are available online at www.mdpi.com/1660-4601/13/3/310/s1, Table S1: Risk factors for diarrhea in children aged 0–23 months in Indonesia (*n* = 4909)—Presentation of prevalence ratio (PR), Table S2: Subgroup analysis on association between diarrhea in children aged less than 24 months and disposal of child’s feces by biological indicators, socio economic indicators and wash indicators—Comparison of odds ratio (OR) and prevalence ratio (PR).

## References

[1] RISKESDAS (2013). National Heath Survey.

[2] WHO/UNICEF (2015). Progress on Sanitation and Drinking Water—2015 Update and MDG Assessment.

[3] UN (2014). Levels & Trends in Child Mortality—2014 Report: Estimates Developed.

[4] RISKESDAS (2007). National Heath Survey.

[5] Fewtrell L., Prüss-Üstün A., Bos R., Gore F., Bartram J. (2007). Water, Sanitation and Hygiene: Quantifying the Health Impact at National and Local Levels In Countries with Incomplete Water Supply and Sanitation Coverage.

[6] Wolf J., Prüss-Ustün A., Cumming O., Bartram J., Bonjour S., Cairncross S., Clasen T., Colford J.M., Curtis V., De France J. (2014). Assessing the impact of drinking water and sanitation on diarrhoeal disease in low- and middle-income settings: Systematic review and meta-regression. Trop. Med. Int. Health.

[7] Government of India (2012). Sanitation and Hygiene Advocacy and Communication Strategy Framework 2012–2017.

[8] Aulia H., Surapaty S.C., Bahar E., Susanto T.A., Hamzah M., Ismail R. (1994). Personal and domestic hygiene and its relationship to the incidence of diarrhoea in South Sumatera. J. Diarrhoeal. Dis. Res..

[9] Sima L.C., Desai M.M., McCarty K.M., Elimelech M. (2012). Relationship between use of water from community-scale water treatment refill kiosks and childhood diarrhoea in Jakarta. Am. J. Trop. Med. Hyg..

[10] Semba R.D., de Pee S., Kraemer K., Sun K., Thorne-Lyman A., Moench-Pfanner R., Sari M., Akhter N., Bloem M.W. (2009). Purchase of drinking water is associated with increased child morbidity and mortality among urban slum-dwelling families in Indonesia. Int. J. Hyg. Environ. Health.

[11] Semba R.D., Kraemer K., Sun K., de Pee S., Akhter N., Moench-Pfanner R., Rah J.H., Campbell A.A., Badham J., Bloem M.W. (2011). Relationship of the presence of a household improved latrine with diarrhoea and under-five child mortality in Indonesia. Am. J. Trop. Med. Hyg..

[12] Cameron L., Shah M., Olivia S. (2013). Impact Evaluation of a Large-Scale Rural Sanitation Project in Indonesia: Impact Evaluation.

[13] Komarulzaman A., Smits J., de Jong E. (2014). Clean Water, Sanitation and Diarrhoea in Indonesia: Effects of Household and Community Factors.

[14] Usfar A.A., Iswarawanti D.N., Davelyna D., Dillon D. (2010). Food and personal hygiene perceptions and practices among caregivers whose children have diarrhoea: A qualitative study of urban mothers in Tangerang, Indonesia. J. Nutr. Edu. Behav..

[15] Agustina R., Sari T.P., Satroamidjojo S., Bovee-Oudenhoven I.M.J., Feskens E.J.M., Kok F.J. (2013). Association of food-hygiene practices and diarrhoea prevalence among Indonesian young children from low socioeconomic urban areas. BMC Pub. Health.

[16] Lanata C.F., Huttly S.R., Yeager B.A. (1998). Diarrhea: Whose feces matter? Reflections from studies in a Peruvian shanty town. Pediatr. Infect. Dis. J..

[17] (2013). BPS (Ministry of Statistics Indonesia), NPaFPBB, MoH, ICF International, Indonesia Demographic and Health Survey. http://dhsprogram.com/what-we-do/survey/survey-display-357.cfm.

[18] WHO (2008). Indicators for Assessing Infant and Young Child Feeding Practices: Part 1.

[19] WSP/UNICEF (2014). Child Feces Disposal in Indonesia.

[20] WSP (2015). Management of Child Feces: Current Disposal Practices.

[21] MoH (2013). Guidance Book on Verification of the STBM Program—Community Based Total Sanitation and Hygiene.

[22] Fink G., Günther I., Hill K. (2011). The effect of water and sanitation on child health: evidence from the demographic and health surveys 1986–2007. Int. J. Epidemiol..

[23] Gil A., Lanata C., Kleinau E., Penny M. (2004). Children’s Feces Disposal Practices in Developing Countries and Interventions to Prevent Diarrheoal Diseases.

[24] Pickering A.J., Davis J. (2012). Freshwater availability and water fetching distance affect child health in sub-Saharan Africa. Environ. Sci. Technol..

[25] Mosites E.M., Rabinowitz P.M., Thumbi S.M., Montgomery J.M., Palmer G.H., May S., Rowhani-Rahbar A., Neuhouser M.L., Walson J.L. (2015). The relationship between livestock ownership and child stunting in three countries in Eastern Africa using national survey data. PLoS ONE.

[26] Sinmegn Mihrete T., Asres Alemie G., Shimeka Teferra A. (2014). Determinants of childhood diarrhea among underfive children in Benishangul Gumuz Regional State, North West Ethiopia. BMC Pediatr..

[27] Siziya S., Muula A.S., Rudatsikira E. (2009). Diarrhoea and acute respiratory infections prevalence and risk factors among under-five children in Iraq in 2000. Ital. J. Pediatr..

[28] George C.M., Oldja L., Biswas S., Perin J., Lee G.O., Kosek M., Sack R.B., Ahmed S. (2015). Geophagy is associated with environmental enteropathy and stunting in children in rural Bangladesh. Am. J. Trop. Med. Hyg..

[29] Ngure F., Humphrey J.H., Mbuya M.N., Majo F., Mutasa K., Govha M., Mazarura E., Chasekwa B., Prendergast A.J., Curtis V., Boor K.J. (2013). Formative research on hygiene behaviors and geophagy among infants and young children and implications of exposure to fecal bacteria. Am. J. Trop. Med. Hyg..

[30] Sodha S.V., Menon M., Trivedi K. (2011). Microbiologic effectiveness of boiling and safe water storage in South Sulawesi, Indonesia. J. Water Health.

[31] Ramli, Agho K.E., Inder K.J., Bowe S.J., Jacobs J., Dibley M.J. (2009). Prevalence and risk factors for stunting and severe stunting among under-fives in North Maluku province of Indonesia. BMC Pediatr..

[32] Howard C.T., de Pee S., Sari M., Bloem M.W., Semba R.D. (2007). Association of diarrhea with anemia among children under age five living in rural areas of Indonesia. J. Trop. Pediatr..

[33] Curtis V., Kanki B., Mertens T., Traore E., Diallo I., Tall F., Cousens S. (1995). Potties, pits and pipes: Explaining hygiene behaviour in Burkina Faso. Soc. Sci. Med..

[34] Yeager B., Huttly S., Bartolini R., Rojas M., Lanata C.F. (1999). Defecation practices of young children in a Peruvian shanty town. Soc. Sci. Med..

[35] Sultana R., Mondal U.K., Rimi N.A., Unicomb L., Winch P.J., Nahar N., Luby S.P. (2013). An improved tool for household faeces management in rural Bangladeshi communities. Trop. Med. Int. Health.

[36] Mellington N., Cameron L. (1999). Female education and child mortality in Indonesia. Bull Indones Econ. Stud..

[37] Agustina R., Shankar A.V., Ayuningtyas A., Achadi E.L., Shankar A.H. (2015). Maternal agency influences the prevalence of diarrhea and acute respiratory tract infections among young Indonesian children. Matern. Child Health J..

[38] Dreibelbis R., Winch P.J., Leontsini E., Hulland K.R., Ram P.K., Unicomb L., Luby S.P. (2013). The integrated behavioural model for water, sanitation, and hygiene: A systematic review of behavioural models and a framework for designing and evaluating behaviour change interventions in infrastructure-restricted settings. BMC Public Health.

[39] Sigler R., Mahmoudi L., Graham J.P. (2015). Analysis of behavioral change techniques in community-led total sanitation programs. Health Promot. Int..

[40] Brown J., Cairncross S., Ensink J.H.J. (2013). Water, sanitation, hygiene and enteric infections in children. Arch. Dis. Child.

[41] Feachem R., Bradley D., Garelick H., Garelick D.M. (1983). Sanitation and Disease: Health Aspects of Excreta and Wastewater Management. World Bank Studies in Water Supply and Sanitation 3.

[42] Al-Gallas N., Bahri O., Bouratbeen A. (2007). Etiology of acute diarrhea in children and adults in Tunis, Tunisia, with emphasis on diarrheagenic *Escherichia coli*: Prevalence, phenotyping, and molecular epidemiology. Am. J. Trop. Med. Hyg..

[43] UNICEF (2014). Sanitation and Hand Washing Baseline and Knowledge, Attitudes and Practices (KAP) Study in Support of the Strengthening Community Approaches to Total Sanitation (STBM) Project in Six Districts of Eastern Indonesia.

[44] WSP (2009). Total Sanitation and Sanitation Marketing Research Report.

[45] Torlesse H., Cronin A.A., Sebayang S.K., Nandy R.K. Determinants of stunting in Indonesian children: evidence from a cross-section survey indicate a role for the water, sanitation and hygiene sector in stunting reduction. BMC Public Health.

[46] Majorin F., Freeman M.C., Barnard S., Routray P., Boisson S., Clasen T. (2014). Child Feces Disposal Practices in Rural Orissa: A Cross Sectional Study. PLoS ONE.

[47] Miller-Petrie M.K., Voigt L., McLennan L. (2015). Infant and young child feces management and enabling products for their hygienic collection, transport, and disposal in in Cambodia. Am. J. Trop. Med. Hyg..

[48] Muluken A., Haile D. (2015). Factors associated with safe child feces disposal practices in Ethiopia: Evidence from demographic and health survey. Arch. Public Health.

[49] Winters W.M., Karim A.G., Martawardya B. (2014). Public Service Provision under Conditions of Insufficient Citizen Demand: Insights from the Urban Sanitation Sector in Indonesia.

[50] Schmidt W.P., Boisson S., Genser B., Barreto M.L., Baisley K., Filteau S., Cairncross S. (2010). Weight-for-age z-score as a proxy marker for diarrhoea in epidemiological studies. J. Epidemiol. Community Health.

[51] Velleman Y., Mason E., Graham W., Benova L., Chopra M., Campbell O.M., Gordon B., Wijesekera S., Hounton S., Esteves Mills J. (2014). From joint thinking to joint action: A call to action on improving water, sanitation, and hygiene for maternal and newborn health. PLoS Med..

[52] WHO/UNICEF (2013). Ending Preventable Child Deaths from Pneumonia and Diarrhoea by 2025: The integrated Global Action Plan for Pneumonia and Diarrhoea (GAPPD).

