# Supplementary material for: Association of Safe Disposal of Child Feces and Reported Diarrhea in Indonesia: Need for Stronger Focus on a Neglected Risk
Source: Int J Environ Res Public Health. 2016 Mar 11;13(3):310. doi: 10.3390/ijerph13030310 (PMC4808973; doi:10.3390/ijerph13030310)
Supplement: Supplementary file 1 [file ijerph-13-00310-s001.pdf]

Supplementary Materials: Association of Safe Disposal of Child Feces and Reported Diarrhea in Indonesia: Need for Stronger Focus on a Neglected Risk

Aidan A. Cronin, Susy Katikana Sebayang, Harriet Torlesse, Robin Nandy

Table S1. Risk factors for diarrhea in children aged 0–23 months in Indonesia (n = 4909)—Presentation of prevalence ratio (PR).

Factors		Diarrhea (%)	No of Cases	N	Unadjusted (bivariate) PR	95% CI		*p*	Adjusted (multivariate) PR	95% CI		*p*
						Lower	Upper			Lower	Upper	
**Sex**	Boys	19.3%	492	2548	1.13	0.94	1.35	0.19				
	Girls	17.1%	404	2362								
**Age of child (months)**	12–23	20.6%	486	2364	1.61	1.27	2.05	0.0005	1.85	1.46	2.35	<0.0001
	6–11	19.2%	253	1321	1.50	1.15	1.97		1.64	1.26	2.12	
	0–5	12.8%	156	1225	Ref							
**Mother's perception of child size at birth**	Very small	43.1%	28	64	3.25	1.55	6.83	0.002	3.06	1.47	6.37	0.007
	Smaller than average	19.8%	108	546	1.50	0.77	2.90		1.49	0.78	2.83	
	Average	18.2%	510	2793	1.38	0.75	2.53		1.39	0.77	2.53	
	Larger than average	17.1%	225	1315	1.29	0.69	2.40		1.32	0.72	2.41	
	Very large	13.2%	25	192	Ref				Ref			
**Age-appropriate feeding**	No	18.1%	562	3106	0.98	0.82	1.16	0.79				
	Yes	18.5%	334	1803	Ref							
**Woman's age**	<20	29.0%	80	277	2.01	1.15	3.50	<0.0001	1.91	1.11	3.30	0.0001
	20–29	20.3%	522	2566	1.41	0.88	2.25		1.38	0.87	2.20	
	30–39	14.1%	263	1857	0.98	0.61	1.58		0.97	0.60	1.56	
	≥40	14.5%	30	209	Ref				Ref			
**Husband's age**	<30	23.7%	369	1562	1.62	1.24	2.12	<0.0001				
	30–39	16.1%	394	2443	1.11	0.86	1.43					
	≥40	14.6%	132	904	Ref							
**Women's education**	Primary or less	19.4%	261	1342	1.62	1.15	2.28	0.01	1.48	1.06	2.06	0.04
	Secondary	19.4%	543	2804	1.61	1.17	2.22		1.50	1.09	2.07	
	Higher	12.0%	92	763	Ref				Ref			
**Husband's education**	Primary or less	20.6%	293	1424	1.73	1.21	2.49	0.01				
	Secondary	18.6%	520	2789	1.57	1.10	2.24					
	Higher	11.9%	83	696	Ref							

Table 1. *Cont.*

Factors		Diarrhea (%)	No of Cases	N	Unadjusted (bivariate) PR	Unadjusted 95% CI Lower	Unadjusted 95% CI Upper	Unadjusted *p*	Adjusted (multivariate) PR	Adjusted 95% CI Lower	Adjusted 95% CI Upper	Adjusted *p*
**Number of household member**	>4	19.1%	594	3114	1.13	0.94	1.36	0.18				
	≤4	16.8%	302	1795	Ref							
**Wealth Quintile**	Lowest	22.9%	150	655	1.68	1.25	2.27	0.014				
	Second	19.8%	189	951	1.46	1.07	1.97					
	Third	19.3%	198	1031	1.41	1.03	1.95					
	Fourth	17.8%	207	1162	1.31	0.94	1.81					
	Highest	13.6%	151	1110	Ref							
**Sanitation**	Unimproved	20.7%	319	1537	1.21	1.01	1.46	0.04				
	Improved	17.1%	577	3372	Ref							
**Safe disposal of child feces**	Unsafe	20.4%	530	2593	1.29	1.09	1.54	<0.0001	1.35	1.14	1.61	0.001
	Safe	15.8%	366	2316	Ref							
**Availability of soap for hand washing**	Unavailable	24.9%	76	307	1.40	1.08	1.81	0.01				
	Available	17.8%	819	4602	Ref							
**Water source**	Unimproved	19.1%	361	1886	1.08	0.91	1.29	0.37				
	Improved	17.7%	535	3023	Ref							
**Water treatment**	Untreated	17.5%	268	1534	0.94	0.78	1.14	0.54				
	Treated	18.6%	627	3375	Ref							
**Number of antenatal care visits received by mother during pregnancy**	<4	22.2%	93	418	1.24	0.98	1.57	0.07				
	≥4	17.9%	803	4492	Ref							
**Birth assisted by trained professionals**	No	20.4%	102	499	1.13	0.90	1.43	0.28				
	Yes	18.0%	794	4410	Ref							
**Birth in facility**	No	19.2%	571	2983	1.14	0.94	1.38	0.19				
	Yes	16.8%	324	1926	Ref							
**Woman participates in decision on own health**	No	24.3%	178	734	1.42	1.13	1.77	0.002	1.32	1.07	1.62	0.009
	Yes	17.2%	717	4175	Ref				Ref			
**Woman participates in decision on large household purchase**	No	22.2%	186	837	1.28	1.04	1.56	0.02				
	Yes	17.4%	709	4072	Ref							
**Woman participates in decision on visit to family**	No	23.1%	172	744	1.33	1.08	1.63	0.01				
	Yes	17.4%	724	4165	Ref							
**Woman participates in decision on what to do with money**	No	21.8%	117	535	1.23	0.95	1.58	0.11				
	Yes	17.8%	779	4374	Ref							

Table 2. Subgroup analysis on association between diarrhea in children aged less than 24 months and disposal of child's feces by biological indicators, socio economic indicators and wash indicators—Comparison of odds ratio (OR) and prevalence ratio (PR).

Subgroup	N	Adjusted (multivariate) *					Adjusted (multivariate) *				
		OR	95% CI		*p*	*Interaction P*	PR	95% CI		*p*	*Interaction p*
			Lower	Upper				Lower	Upper		
**Biological Indicator**											
**Gender** *											
Male	2548	1.15	0.85	1.56	0.37	0.02	1.12	0.88	1.41	0.37	0.02
Female	2362	1.93	1.41	2.65	<0.0001		1.70	1.31	2.20	0.00	
**Birth Size** **											
Smaller than Average	610	2.31	1.27	4.22	0.006	0.12	1.90	1.19	3.04	0.01	0.13
Average to Very Large	4299	1.38	1.09	1.74	0.007		1.29	1.07	1.56	0.01	
**Socio-Economic Indicator**											
**Woman's education** ***											
secondary or lower	4146	1.52	1.20	1.92	<0.0001	0.28	1.39	1.15	1.67	0.00	0.33
Higher	763	1.08	0.62	1.89	0.79		1.07	0.66	1.74	0.79	
**Wash Indicator**											
**Sanitation** *											
Unimproved	1537	2.19	1.40	3.43	0.001	0.04	1.86	1.28	2.69	0.00	0.04
Improved	3372	1.24	0.94	1.62	0.12		1.18	0.95	1.47	0.12	
**Water Source** *											
Unimproved water	1886	1.58	1.12	2.23	0.009	0.57	1.44	1.09	1.89	0.01	0.58
Improved water	3023	1.39	1.06	1.84	0.018		1.30	1.04	1.62	0.02	
**Soap Availability** *											
No	307	1.32	0.61	2.83	0.479	0.81	1.22	0.69	2.16	0.50	0.74
Yes	4602	1.45	1.16	1.82	0.001		1.35	1.12	1.61	0.00	

* Adjusted for woman's education, child's age, birth size, woman's age, and woman participation in decision of her own health; ** Adjusted for woman's education, child's age, woman's age, and woman participation in decision of her own health; *** Adjusted for child's age, birth size, woman's age, and woman participation in decision of her own health.

© 2016 by the authors; licensee MDPI, Basel, Switzerland. This article is an open access article distributed under the terms and conditions of the Creative Commons by Attribution (CC-BY) license (http://creativecommons.org/licenses/by/4.0/).
